# Effect of malting and fermentation on colour, thermal properties, functional groups and crystallinity level of flours from pearl millet (*Pennisetum glaucum*) and sorghum (*Sorghum bicolor*)

**DOI:** 10.1016/j.heliyon.2020.e05467

**Published:** 2020-12-07

**Authors:** G. Olamiti, T.K. Takalani, D. Beswa, A.I.O. Jideani

**Affiliations:** a Department of Food Science and Technology, School of Agriculture, University of Venda, Private Bag X5050, Thohoyandou 0950, South Africa; b Department of Biotechnology and Food Technology, Faculty of Science, University of Johannesburg, P.O. Box 17011, Doornfontein 2028, Johannesburg, South Africa

**Keywords:** Food science, Food analysis, Fermentation, Malting, Sorghum, Pearl millet, Colour, Thermal properties, XRD, FTIR

## Abstract

The effect of malting and fermentation on colour, thermal properties, level of crystallinity and functional groups of *Agrigreen*, *Babala* pearl millet cultivars and sorghum flours were studied using response surface methodology. The central composite rotatable design was performed on two independent variables in terms of malting and fermentation time at intervals of 24, 48 and 72 h, respectively using design expert software. One-way analysis of variance at p < 0.05, regression analysis, response surface plots for interactions between malting and fermentation processing times with response variables were recorded. The results indicated that malting and fermentation times have significant effects on the thermal and colour properties as well as the level of crystallinity and functional groups of pearl millet cultivars and sorghum flours. In terms of colour, sorghum exhibited high content in L∗ at 72.02–73.72, a∗ ranged from 2.50-3.30 and chrome at 13.10–14.82, while *Babala* flour was high in b∗ and hue at 12.15–14.27 and 73.00–84.80, respectively. In terms of thermal properties, sorghum was noticed to be high in melting peak at 87.57–104.83 °C, 102.66–111.14 °C for end completion and gelatinisation range at 10.70–25.79 °C, whereas, *Babala* recorded high values in onset and enthalpy at 93.20–100.11 and 5.72–21.62 J/g, respectively. The Fourier transform infrared (FTIR) spectroscopy showed that malted and fermented *Agrigreen, Babala* and sorghum flour showed peaks in OH, carbonyl, amide and C–O bonding. The optimal processing time for the colour of *Agrigreen* was 50.69 h (malting) and 39.38 h (fermentation), *Babala* was 54.40 h (malting) and 65.30 h (fermentation); and sorghum was 49.90 h (malting) and 54.61 h (fermentation). While the optimal malting and fermentation time for thermal properties for *Agrigreen* was 45.78 h and 42.60 h; *Babala* was 40.94 h and 29.07 h and sorghum was 34.83 h and 36.33 h, respectively with product quality at the desirability of 1.00. X-ray diffractogram results of the optimum processing points of the thermal properties showed that malted and fermented *Agrigreen*, *Babala* and sorghum flour showed high peak intensities, while the unprocessed flour exhibited diffused peaks. The obtained results would assist food processing companies to improve the colour and thermal properties and also the behaviour of the crystallinity and functional groups in food during processing.

## 1 Introduction

Pearl millet (*Pennisetum glaucum*) and sorghum (*Sorghum bicolor*) are grain crops that are planted in the dry and semi-dry part of Africa (Eltayeb et al., 2007; Shahidi and Chandrasekara, 2013). These crops are nutritionally high and considered underutilised crops (Taylor and Duodu, 2015) in which it's nutritional and nutraceutical benefits have not been sufficiently utilised. Pearl millet and sorghum grains contain a high percentage of nutritional (protein, fatty acids, vitamins) and bioactive compounds (hydroxybenzoic and hydroxycinnamic acids derivatives) (Gong et al., 2018; Girard and Awika, 2018) which are hindered from bioaccessibility because of antinutritional factors present in them, which might affect the thermal digestibility of starch and colour changes during processing. These antinutritional contents are reduced during processing either by leaching or through enzymatic activities on the bioactive compounds such as carbohydrate, protein, and fats in pearl millet and sorghum (Hassan et al., 2006; Eltayeb et al., 2007; Wang et al., 2014) which equally change the colour of the flour.

Malting and fermentation are the important unit operations used for the improvement of the quality of food during processing. Malting of cereal grains is the process of changing the biochemical modifications that improve its nutritional and bioactive quality under controlled germination (Dahiya et al., 2018). Khoddami et al. (2017) described that malting affects the colour properties of sorghum flour. Considering the potential of malting in brewing, the study of Pelembe et al. (2001) reported that pearl millet and sorghum showed improvement in β-amylase and free α-amino nitrogen, showing good indexes for beer production. During malting, endogenous enzymes hydrolyze starch thereby breaking it into smaller molecular weights. Malting increases total sugar and free fatty acids because of protease and amylase enzymes, which break the complex formation of protein and carbohydrates into simple and soluble pieces in cereals (Sandberg and Andlit, 2002). Saleh et al. (2013) and Archana and Kawatra (2001) stated that malting of pearl millet increased the total sugar and *in vitro* protein and starch digestibility. This improvement in protein and starch digestibility could be credited to the reduction in antinutrients present in the cereal grains forming complexes with the protein (Hassan et al., 2006). Fermentation is a process where microorganism enzymes act on a substrate especially a carbohydrate, which releases energy, acids, gas, and alcohol (Kohajodova and Karovicova, 2007). Taylor and Duodu (2015) reported lactic acid bacteria (LAB) as the common food fermentation process. LAB fermentation in food reduces the effect of antinutritional content through the assistance of *Lactobacillus* species such as *Lactobacillus acidophilus* (Hurrel, 2004). Fermentation has great importance in improving the nutritional compositions of food and its preservation. This method assists in the preservation of food products while helping to improve the flavour, colour and subsequently the nutritional values of the raw materials (Chinenye et al., 2017; Saleh et al., 2013). Likewise, the study of Hassan et al. (2006) described that fermentation of pearl millet decreases the antinutritional content in food grains and equally increases the protein availability, in-vitro protein, and starch digestibility and consequently the nutritional composition of the grains. Similarly, Ahmed et al. (2009) and Kazanas and Fields (2006) highlighted that the fermentation of sorghum improved chemical compositions such as moisture, ash, fibre, and protein.

Processing methods assist in the modification of the functional groups in the starch molecules, where such modification alters gelatinisation, pasting and retrogradation behaviour of flour and starch (Singh and Sandhu, 2007). This modification on the functional group of starch during processing is determined by the ratio of amylose to amylopectin. The onset (*T*_*o*_), melting temperature (*T*_*p*_), completion temperature (*T*_*c*_), gelatinisation range and enthalpy (J/g) parameters are determined based on their contributions to the level of its gelatinisation potentials (Anyasi et al., 2017). Ahmed et al. (2016) reported the thermal properties of sorghum starch. Sozer et al. (2007); Jideani and Scott (2012) equally reported the thermal and textural characteristics of processed pearl millet. Adebiyi et al. (2016) reported the influence of processing such as malting and fermentation of flour on the crystallinity level and functional groups of pearl millet flour. Similarly, the influence of processing on the colour qualities of pearl millet was reported by Rani et al. (2018). From literature, there is a dearth of information on the usage of mathematical models to explain a combined unit operation of pearl millet cultivars especially the *Agrigreen* and *Babala* species produced in South Africa. This study investigates the results of malting and fermentation on the colour, thermal properties, the level of crystallinity and functional groups of processed pearl millet cultivars and sorghum flour.

## 2 Material and methods

Pearl millet (*Pennisetum glaucum*) cultivars such as *Agrigreen* and *Babala* were purchased from Agricol Pretoria, South Africa. *Sorghum* (*Sorghum bicolor*) grains used as a reference for the study were bought from Thohoyandou market, Limpopo province, South Africa.

### 2.1 Malting

Malting of grains was perfomed according to Eltayeb et al. (2007) and Nithya et al. (2007). The grains of pearl millet cultivars and sorghum were sorted, cleaned and immersed in distilled water for 6 h. The grains were drained and washed twice with formaldehyde to reduce the growth of microorganisms. The grains were later spread on stainless trays lined with muslin wet cloth. The grains were watered 2–3 times in a day and germinated at a controlled temperature at 25 °C for 24 h, 48 h, and 72 h, respectively in an incubator. The malted grains were dried in the oven dryer (Prolab Instrument, South Africa) at 50 °C for 10 h. Dried grains were kept in airtight polyethylene 200 × 250mm resealable bags for further usage.

### 2.2 Fermentation

The fermentation of grains of sorghum and pearl millet cultivars was performed according to El-Tinay et al. (1979) and Fasasi (2009). Grains of sorghum and pearl millet cultivars were fermented naturally by lactic acid bacteria for 24 h, 48 h, and 72 h under a controlled temperature of 25 °C. The grains were rinsed with distilled water and dried in an oven dryer (Prolab Instrument, South Africa) at 50 °C for 10 h. The dried grains were milled (Retsech ZM 200 Miller, Haan, Germany) at 16,000 rpm for 1 min and sieved using 450 μm mesh to obtain fermented flour. The obtained fermented flour was kept in airtight polyethylene bags (200 × 250 mm resealable) and stored in a cool dry place for further use.

### 2.3 Colour determination

The colour of malted and fermented flours of pearl millet and sorghum was determined using a colourimeter (Lovibond LC 100 Spectrocolorimeter, England). The colour of the malted and fermented flour was expressed as L∗- value (lightness) (+) and darkness (-); a∗- value (redness (+) and greenness (-)); b∗- value (yellowness (+) and blueness (-); chroma and hue according to Thuwapanichayanan et al. (2011). The measurements were performed in triplicates and the mean results were reported mean ± standard deviation.

### 2.4 Determination of thermal properties of flour

Thermal properties of malted and fermented flours of pearl millet cultivars and sorghum were evaluated according to the method of Escamilla-Silva et al. (2003) using a Perkin-Elmer DSC (Model DSC 4000). Approximately 25 mg of flour was weighed to the nearest 0.01 mg on a DSC stainless steel pans and scanned at a heating rate of 10 ⁰C/min from 30 to 130 °C. Onset, peak, concluding temperature, and gelatinisation enthalpy were measured and recorded using the Pyris thermal system software.

### 2.5 X-ray diffraction (XRD) analysis of flour

The crystallinity property of the flour was determined according to Adebiyi et al. (2016). The flours of pearl millet and sorghum were ground and sieved to particle sizes 40 μm. A total of 5 g of the sieved flour samples were loaded into the XRD sample holder and pressed down using a stainless steel weight. The level of crystalline property and X-ray diffraction of the flours were determined using an X-ray diffractometer (Rigaku – UltimalV, Japan) equipped with a divergence slit, operating at 40 kV and 40 mA and scanning region was 5–90 °C at a scan speed of 2 °C/min, which covered all the significant sample crystallites (Adebiyi et al., 2016).

### 2.6 Fourier transform-infrared (FTIR) spectroscopy of flour

The functional groups of the flours of pearl millet and sorghum were determined according to Adebiyi et al. (2016). The FTIR spectra of the flours were obtained using an FTIR spectrophotometer [Thermo Scientific Smart iTR, (Attenuated Total Reflectance), Thermo Fisher Scientific Inc. USA]. Approximately 0.5 g of flour was placed on the instrument and the spectra were determined with distinctive peaks in wavenumbers from 450 to 4000 cm^−1^ at 16 runs per scan.

### 2.7 Experimental design

Two independent variables (malting (X_1_) and fermentation (X_2_)) were studied for colour, thermal properties, crystallinity level and functional groups of *Agrigreen, Balaba* and sorghum flour. Response variables for colour (L∗, a∗, b∗ ⁰hue, and chrome) and thermal properties such as onset, peak melting, end completion, gelatinisation range, and enthalpy were measured using Eq. (1). A central composite rotatable design (CCRD) was adopted (Myers and Montgomery, 2002). Using the platform provided for two-variable cases, thirteen experimental runs were carried out with five replications of centre points. The two levels of each of these independent variables were shown in Table 1 with coded and the actual values. A polynomial regression model was expected for predicting individual Y responses.

(1) Y = β_0_ + β_1_X_1_ + β_2_X_2_ + β_12_X_1_X_2_ + β_11_X_12_ + β_22_X_22_ + ε

where: Y = predicted response, β_0_ model constant, X_1_ and X_2_ = independent variables; β_1_ and β_2_ = linear coefficients; β_12_ = cross product coefficient and β_11_ and β_22_ = quadratic coefficients.

**Table 1:** Central composite rotatable design for the independent variables.

Independent variable	Variable	Coded and real values		
		-1	0	+1
Malting (h)	malting time (h) [X_1_]	24.00	48.00	72.00
Fermentation (h)	fermentation time (h) [X_2_]	24.00	48.00	72.00

### 2.8 Statistical analysis

All the experimental techniques were carried out in triplicate and results recorded as a mean ± standard deviation. Design-Expert 11 (Statease Inc; Minneapolis USA, version) was used to process the collected data. Analysis of variance (ANOVA) at p < 0.05, regression analysis and response surface plots were generated for different interactions for malting and fermentation times (Omolola et al., 2015; Akinoso and Adeyanju, 2012).

## 3 Results and discussion

### 3.1 Effect of malting and fermentation time on colour of pearl millet and sorghum flours

The effect of malting and fermentation time on colour of pearl millet cultivars and sorghum flours under processing conditions show that L∗ values for *Agrigreen*, *Babala* cultivars and sorghum were ranged from 63.40-67.39 (Table 2); 65.24–70.92 (Table 3) and 72.02–73.72 (Table 4), respectively. After malting and fermentation times, sorghum flour exhibited a white colour change, indicating a high L∗ value at 24 h malting and 72 h fermentation time compared to *Agrigreen* (48h malting and 81.9h fermentation) and *Babala* (48 h malting and 81.89 h fermentation) flours with less level of L∗. From the results, it was evident that an increase in malting and fermentation time increased the lightness of the flour, while a decrease in malting and fermentation times reduced the lightness of the flour. The obtained results of L∗ were within the range of soaked finger millet flour reported by Ramashia et al. (2018), but higher than 64.61 recorded for roasted pearl millet (Mridula et al., 2008) and 68.4 recorded for finger millet by Siwela et al. (2007). The reason for the high level of L∗ could be attributed to the leaching of phytochemicals during malting and the specie of the grains.

**Table 2:** Effect of malting and fermentation time on colour of *Agrigreen* pearl millet flour.

Runs	Independent variables		Response variables				
	Malting X_1_ (h)	Fermentation X_2_ (h)	L∗	a∗	b∗	⁰hue	chroma
1	48.00	48.00	66.75 ± 0.33	5.15 ± 0.63	12.95 ± 0.07	68.40 ± 0.45	13.77 ± 0.90
2	24.00	24.00	63.40 ± 0.25	5.70 ± 0.14	12.56 ± 0.22	65.57 ± 0.15	13.83 ± 0.20
3	48.00	48.00	66.75 ± 0.33	5.15 ± 0.63	12.95 ± 0.07	68.40 ± 0.45	13.77 ± 0.90
4	48.00	48.00	66.75 ± 0.33	5.15 ± 0.63	12.95 ± 0.07	68.40 ± 0.45	13.77 ± 0.90
5	24.00	72.00	65.35 ± 0.49	5.62 ± 0.25	12.63 ± 0.12	66.03 ± 0.81	13.79 ± 0.26
6	48.00	48.00	66.75 ± 0.33	5.15 ± 0.63	12.95 ± 0.07	68.40 ± 2.45	13.77 ± 0.90
7	48.00	48.00	66.75 ± 0.33	5.15 ± 0.63	12.95 ± 0.07	68.40 ± 2.45	13.77 ± 0.90
8	72.00	72.00	66.38 ± 0.01	5.20 ± 0.34	13.02 ± 0.68	68.15 ± 0.19	13.95 ± 0.49
9	14.06	48.00	66.87 ± 0.04	5.40 ± 0.28	13.65 ± 0.91	68.39 ± 0.43	14.80 ± 0.57
10	48.00	14.06	66.58 ± 0.04	5.25 ± 0.78	14.00 ± 0.56	69.62 ± 0.73	14.67 ± 0.17
11	81.94	48.00	66.71 ± 0.01	5.14 ± 0.01	13.88 ± 0.01	69.30 ± 0.28	14.70 ± 0.28
12	72.00	24.00	64.42 ± 0.70	5.28 ± 0.45	12.95 ± 0.78	67.69 ± 0.84	13.10 ± 0.43
13	48.00	81.94	67.39 ± 0.44	5.40 ± 0.99	12.95 ± 0.07	67.54 ± 0.67	13.77 ± 0.90

L- value (lightness), a-value (redness), b-value (yellowness).

**Table 3:** Effect of malting and fermentation time on colour of *Babala* pearl millet flour.

Runs	Independent variables		Response variables				
	Malting X_1_ (h)	Fermentation X_2_ (h)	L∗	a∗	b∗	⁰hue	chroma
1	48.00	48.00	67.40 ± 0.40	1.52 ± 0.17	12.70 ± 0.84	73.30 ± 0.57	13.00 ± 0.57
2	24.00	24.00	67.52 ± 0.24	1.10 ± 0.00	12.15 ± 0.63	84.80 ± 0.14	12.20 ± 0.71
3	48.00	48.00	67.4 0 ± 0.40	1.52 ± 0.17	12.70 ± 0.84	73.30 ± 0.57	13.00 ± 0.57
4	48.00	48.00	67.40 ± 0.40	1.52 ± 0.17	12.70 ± 0.84	73.30 ± 0.57	13.00 ± 0.57
5	24.00	72.00	69.24 ± 0.62	1.04 ± 0.09	12.30 ± 0.42	85.17 ± 0.37	12.37 ± 0.47
6	48.00	48.00	67.40 ± 0.40	1.52 ± 0.17	12.70 ± 0.84	73.30 ± 0.57	13.00 ± 0.57
7	48.00	48.00	67.40 ± 0.40	1.52 ± 0.17	12.70 ± 0.84	73.30 ± 0.57	13.00 ± 0.57
8	72.00	72.00	67.92 ± 0.24	1.79 ± 0.15	13.90 ± 0.69	83.07 ± 0.34	14.02 ± 0.80
9	14.06	48.00	65.24 ± 0.66	1.62 ± 0.30	13.35 ± 1.77	83.23 ± 0.54	13.45 ± 1.77
10	48.00	14.06	67.05 ± 0.90	1.67 ± 0.04	14.27 ± 0.37	73.34 ± 0.62	14.34 ± 0.32
11	81.94	48.00	64.95 ± 0.60	2.00 ± 0.85	14.15 ± 0.90	81.80 ± 0.55	14.26 ± 0.03
12	72.00	24.00	66.49 ± 0.78	1.85 ± 0.06	13.75 ± 0.89	82.70 ± 0.83	13.85 ± 0.04
13	48.00	81.94	70.92 ± 0.57	1.72 ± 0.11	13.90 ± 0.84	73.00 ± 0.14	14.00 ± 0.84

L- value (lightness), a-value (redness), b-value (yellowness).

**Table 4:** Effect of malting and fermentation time on colour of Sorghum flour.

Runs	Independent variables		Response variables				
	Malting X_1_ (h)	Fermentation X_2_ (h)	L∗	a∗	b∗	⁰hue	chroma
1	48.00	48.00	72.47 ± 0.42	2.70 ± 0.57	13.39 ± 0.83	78.45 ± 0.04	13.72 ± 0.59
2	24.00	24.00	73.20 ± 0.14	2.60 ± 0.99	13.75 ± 0.78	79.15 ± 0.60	14.00 ± 0.57
3	48.00	48.00	72.47 ± 0.42	2.70 ± 0.57	13.39 ± 0.83	78.45 ± 0.04	13.72 ± 0.59
4	48.00	48.00	72.47 ± 0.42	2.70 ± 0.57	13.39 ± 0.83	78.45 ± 0.04	13.72 ± 0.59
5	24.00	72.00	73.77 ± 0.94	2.54 ± 1.08	14.05 ± 0.20	79.59 ± 0.21	14.37 ± 0.09
6	48.00	48.00	72.47 ± 0.42	2.70 ± 0.57	13.39 ± 0.83	78.45 ± 0.04	13.72 ± 0.59
7	48.00	48.00	72.47 ± 0.42	2.70 ± 0.57	13.39 ± 0.83	78.45 ± 0.04	13.72 ± 0.59
8	72.00	72.00	72.77 ± 0.35	3.19 ± 0.00	13.15 ± 0.47	75.65 ± 0.78	13.72 ± 0.00
9	14.06	48.00	72.42 ± 0.35	2.75 ± 0.63	14.54 ± 0.80	79.37 ± 0.75	14.82 ± 0.97
10	48.00	14.06	73.14 ± 0.52	2.60 ± 0.71	9.35 ± 0.26	79.45 ± 0.45	14.60 ± 0.98
11	81.94	48.00	72.02 ± 0.21	3.30 ± 0.41	12.97 ± 0.41	75.42 ± 0.33	13.42 ± 0.01
12	72.00	24.00	72.24 ± 0.51	3.25 ± 0.91	12.85 ± 0.05	75.22 ± 0.16	13.35 ± 0.48
13	48.00	81.94	73.69 ± 0.29	2.50 ± 0.85	13.72 ± 0.30	79.51 ± 0.54	13.10 ± 0.07

L- value (lightness), a-value (redness), b-value (yellowness).

The results of a∗ were ranged; 5.14–5.70, 1.04–2.00 and 2.50–3.30 for *Agrigreen*, *Babala* and sorghum flours, respectively. There was a significant difference in colour on the processed flour at p < 0.05. During processing (malting and fermentation times), *Agrigreen* flour turned to a red colour indicating a high level of a∗ at 24 h malting and 24 h fermentation time, compared to *Babala* (81.94 h malting and 48 h fermentation time) and sorghum (81.94 h malting and 48 h fermentation time) that exhibited less content of a∗. This presence of colour change especially a∗ could be credited to the occurrence of phenolic compounds like tannin present at the pericarp and testa of the grain, which were reduced by leaching (during mating) and polyphenol oxidase during fermentation. These phenolic compounds increase in malting and fermentation time increases the level of a∗ in *Agrigreen* flour, while *Babala* and sorghum recorded a low level of a∗ upon an increase in malting and fermentation time. An increase in a∗ of *Agrigeen* could be a result of the presence of pigment. The obtained result is lower than 5.44 reported for roasted pearl millet by Mridula et al. (2008) and higher than 3.77 reported for finger millet flour by (Ramashia et al., 2018).

The degree of yellowness (b∗) was recorded for all the processed flour where *Agrigreen* ranged from 12.63 -14.00, *Babala* was 12.15–14.27 and sorghum were 9.35–14.05. During processing, it was noticed that malting and fermentation of *Babala* flour had a yellow colour, indicating a high level of yellowish colour (b∗) at 48 h malting and 48 h fermentation time compared to *Agrigreen* (48 h malting and 14 h fermentation time) and sorghum (14 h malting and 48 h fermentation time) with less content of b∗. It was arguable that malted and fermented *Babala* flour had a higher value of b∗ than other processed flour which was influenced by the Increase in malting and fermentation time. The b∗ result of malted and fermented *Babala* pearl millet flour is closer to 12.18 of roasted pearl millet reported by Mridula et al. (2008) and 13.10 for soaked finger millet flour reported by Ramashia et al. (2018), but less than the results of processed sorghum (Afify et al., 2015). Clarifying a∗ and b∗ coordinates, the indication of positive values for malted and fermented *Agrigreen* (a∗) and *Babala* (b∗) flours showed red and yellow pigmentation for the flour.

The hue angle (h^0^) values for malted and fermented flours were recorded in range. *Agrigreen* flour results ranged from 65.57 – 69.62, *Babala* flour was 73.0–84.80 and sorghum flour from 75.22 – 79.51. The obtained results showed a significant difference in the colour of malted and fermented flour at p < 0.05. From the results, it could be inferred that the hue of malted and fermented *Babala* pearl millet cultivar recorded high value at 24 h malting and 72 h fermentation time than malted and fermented *Agrigreen* (48 h and 14 h) and sorghum (24 h and 72 h) flours respectively. The hue of malted and fermented *Babala* pearl millet flour was higher than 77.3 reported for milky cream finger millet cultivar (Ramashia et al., 2018) and 67.25 for roasted pearl millet (Mridula et al., 2008) but lower than the results of Afify et al. (2015) for the processed sorghum. Hue angle is considered as the qualitative quality of colour which is traditionally based on reddish, greenish and others. Ramashia et al. (2018) reported that the hue angle is most important to humans with a usual colour vision for perception and acceptability.

The chroma values of the malted and fermented flour of *Agrigreen*, *Babala* and sorghum flour were recorded in ranges. *Agrigreen* ranged from 13.10 -14.70, 12.20–14.34 for *Babala* and sorghum flour ranged from 13.10 – 14.82. Chroma showed a non-significant difference in the malted and fermented flour.

Chroma of malted and fermented sorghum flour was noticed high at 14.06 h malting and 48 h fermentation time than the processed flour of *Agrigreen* and *Babala* pearl millet cultivars. This implies that all the processed flour exhibited relatively pigment concentration. An increase in chroma could have resulted in the high content of pigment concentration and the colour becomes darker as the concentration reduces. Wrolstad and Smith (2010) argued there could be similarity in food samples in term of hue and chroma, but would only be distinguished using their L∗. Pathare et al. (2013) reported that the higher the chroma values produces high colour intensity of the flour which is perceived by humans. Colour is thus noted as an essential quality parameter in the food processing industry that drives the consumer's choice and preferences. It could be deduced that variations in colour characteristics on processed *Agrigreen*, *Babala* and sorghum flours could be attributed to biochemical processes, leaching of polyphenol during malting and fermentation processing time and the varietal changes in cultivars (Taylor and Duodu, 2015).

The analysis of variance (ANOVA) of the response surface model for the colour of pearl millet and sorghum flours (L∗, a∗, b∗, hue and chroma) were significantly affected at p < 0.05 by different model factors i.e X_1_ (malting time), X_2_ (fermentation time), X_1_ X_2_ (interaction between malting and fermentation), X_1_^2^ (second-order of malting time), and X_2_^2^ (second-order of fermentation time) (Table 5.). Hayate et al. (2014) reported that the extent of the significance of each model parameter was determined by F-value (i.e the greater the F-value of a parameter, the greater the significance). For *Agrigreen* flour, malting time (X_1_), second-order malting (${\text{X}}_{1}^{2}$) and second-order fermentation time (${\text{X}}_{2}^{2}$) showed high significance on a∗. In terms of *Babala*, mating time (${\text{X}}_{1}^{2}$) and (X_1_), were significant in L∗, a∗, b∗ and chroma, while fermentation (X_2_ and ${\text{X}}_{2}^{2}$) were most significant in L∗. For sorghum, model parameters X_1_ and ${\text{X}}_{1}^{2}$ were most significant in L∗, a∗, hue and chroma, while fermentation time (X_2_^2^) had most significant in L∗ and b∗. The processed flour (*Agrigeen*, *Babala* and sorghum) showed linear interactions of malting and fermentation on colour (X_1_ and X_2_), interaction effects of malting and fermentation on colour (X_1_X_2_), quadratic effects of malting and fermentation on colour (${\text{X}}_{1}^{2}$ and ${\text{X}}_{2}^{2}$). This implies that an increase in malting and fermentation influenced the colour characteristics of the processed flour.

**Table 5:** ANOVA results of the effect of malting and fermentation on colour characteristics of *Agrigreen, Babala* pearl millet and sorghum flours.

Source	L∗		a∗		b∗		Hue		Chroma	
	F_value_	P_value_	F_value_	P_value_	F_value_	P_value_	F_value_	P_value_	F_value_	P_value_
***Agrigreen* pearl millet flour**										

Model	1.51	0.2666∗∗	5.190	0.0262∗	0.54	0.7452∗∗	1.96	0.1914∗∗	0.63	0.6834∗∗
X_1_	0.35	0.5681∗∗	13.92	0.0074∗	0.62	0.4581∗∗	3.46	0.0926∗∗	0.23	0.6441∗∗
X_2_	2.68	0.1328∗∗	0.026	0.8766	0.91	0.3712∗∗	0.46	0.5119∗∗	0.099	0.7627∗∗
${\text{X}}_{1}^{2}$			5.110	0.0582∗	1.12	0.3244∗∗			2.09	0.1916∗∗
${\text{X}}_{2}^{2}$			8.380	0.0232∗	0.085	0.7791∗∗			0.011	0.9198∗∗
X_1_X_2_			0.000	1.000	0.000	1.000∗∗			0.73	0.4215∗∗

***Babala* pearl millet flour**										

Model	15.61	0.0011∗	7.590	0.0099∗	2.23	0.1628∗∗	5.03	0.0284∗	3.68	0.0635∗∗
X_1_	2.93	0.1309∗∗	15.18	0.0030∗	5.62	0.0496∗	0.45	0.5231∗∗	7.35	0.0219∗
X_2_	25.57	0.0011∗	8.885e-003	0.9268∗∗	0.017	0.8997∗∗	7.835e-004	0.9785∗∗	7.374e-003	0.9333∗∗
${\text{X}}_{1}^{2}$	19.89	0.0029∗			2.20	0.1818∗∗	24.40	0.001∗		
${\text{X}}_{2}^{2}$	20.56	0.0027∗			3.98	0.0862∗∗	1.38	0.2785∗∗		
X_1_X_2_	0.065	0.8066∗∗			0.000	1.0000∗∗	0.000	1.000∗∗		

**Sorghum flour**										

Model	5.22	0.0258∗	22.8	0.0004∗	4.60	0.0352∗	14.26	0.0015∗	5.33	0.0265∗
X_1_	9.42	0.0181∗	76.32	<0.0001∗	1.95	0.2057∗∗	58.17	0.0001∗	10.15	0.0097∗
X_2_	1.79	0.2232∗∗	0.25	0.6294∗∗	0.043	0.8410∗∗	0.31	0.5947∗∗	0.52	0.4887∗∗
${\text{X}}_{1}^{2}$	0.18	0.6876∗∗	32.99	0.0007∗	2.89	0.1329∗∗	10.81	0.0133∗		
${\text{X}}_{2}^{2}$	14.0.6	0.0072∗	0.85	0.3875∗∗	16.01	0.0052∗	0.95	0.3632∗∗		
X_1_X_2_	4.724e-003	0.9471∗∗	0.000	1.000∗∗	0.000	1.000∗∗	6.425e-005	0.9938∗∗		

∗Significant at p < 0.05, ∗∗ Non-significant at p > 0.05; X_1_- linear effect of malting, X_2_- linear effect of fermentation, X_1_X_2_- the interaction of malting and fermentation, ${\text{X}}_{1}^{2}$ – quadratic effect of malting; ${\text{X}}_{2}^{2}$ – quadratic effect of fermentation. L∗ - (lightness), a∗ - (redness), b∗ - (yellowness) and chroma – Intensity of the colour.

Regression models relating to colour characteristics such as L∗, a∗, b∗, hue and chroma to the independent variables, that is malting and fermentation for *Agrigreen*, *Babala* cultivars of pearl millet and sorghum flours were shown in Table 6. All the processed flour (*Agrigreen*, *Babala*, and sorghum) exhibited a positive intercept for L∗, a∗, b∗, hue, and chroma. Meaning that malting (X_1_) and fermentation (X_2_) time had a positive influence on L∗, a∗, b∗, hue, and chroma. The influence of malting and fermentation on the colour characteristics of processed flour could be deduced as positive or negative. For the processed flour of *Agrigreen* cultivar, L∗ and hue angle showed a linear relationship during malting (X_1_) and fermentation (X_2_). Malting exhibited the most quadratic effects on L∗, b∗ and chroma, while, fermentation recorded most quadratic effects on a∗, b∗ and chroma. Colour parameters of *Agrigeen* pearl flour, a∗ exhibited a high coefficient of variation (R^2^) of 0.787. The higher the coefficient of variation best explains the correlation between the processing variables such as L∗, a∗, b∗, hue and chroma for *Agrigreen* flour. In respect of *Babala*, malting (X_1_) exhibited the most linear effect on a∗ and chroma, while fermentation (X_*2*_) showed the most linear effect on L∗. The most quadratic effect of malting was noticed in b∗ and hue, while L∗, b∗ and hue had the most quadratic effect on the fermentation processing method. Sorghum showed high malting with a linear relationship on b∗ and hue, while a∗, b∗ and chroma exhibited the most linear effect upon fermentation. The most quadratic effect of b∗ was noticed in malting, while L∗ and hue showed the most effect on fermentation. The coefficient of determination (R^2^) of models was relatively high especially for L∗ of *Babala* flour (0.9177) and a∗ of sorghum with 0.941. Having low r^2^ values in some models does not mean that the models are not insignificant. This could be a result of many data points regenerated indicating that little variation in the dependent variables can be explained by variation in the independent variables. A non–significant lack of t-test is good as this strengthens the fitness of the models. Omolola et al. (2015) reported that the coefficient of variation of the parameters indicates the extent and significance of each model parameter with regards to their effects on the response variables. The higher the coefficient of variation of a model parameter, the higher the significance of such parameter as emphasised.

**Table 6:** Regression models of colour response and independent variables for *Agrigreen*, *Babala* pearl millet and sorghum flours.

Response variables	Models	Residual fit at P-value	R^2^
***Agrigreen* pearl millet flour**			

L∗	+64.49942 + 9.499857E-003X_1_ +0.02633X_2_	0.266	0.4323
a∗	+6.33389–0.02264X_1_ -0.020666X_2_+1.70356E-004X_1_^2^ + 2.18099E-004${\text{X}}_{2}^{2}$ + 3.57486-018X_1_X_2_	0.026∗	0.7874
b∗	+14.02991–0.027577X_1_-0.016171X_2_ + 3.4722E-004${\text{X}}_{1}^{2}$ + 9.54861E-005${\text{X}}_{2}^{2}$ + 1.52752E-017X_1_X_2_	1.960	0.4816
Hue	+67.14597 + 0.028786X_1_ – 0.010529X_2_	1.96	0.4816
Chroma	+16.17855–0.069851X_1_-0.024390X_2_ + 4.95877E-004${\text{X}}_{1}^{2}$ + 3.58073E-005${\text{X}}_{2}^{2}$ + 3.86285E-004X_1_X_2_	0.63	0.4108

***Babala* pearl millet flour**			

L∗	+65.70928 + 0.11248X_2_ -1.674226E-003${\text{X}}_{1}^{2}$ + 1.70247E-003${\text{X}}_{2}^{2}$ – 1.25868E-004X_1_X_2_	0.0011∗	0.9177
a∗	+1.07143 + 0.010611X_1_ – 2.56715E-004X_2_	0.0099∗	0.6030
b∗	+14.92866–0.035476X_1_ – 0.077309X_2_ + 5.89193E-004${\text{X}}_{1}^{2}$ + 7.93186E004${\text{X}}_{2}^{2}$ + 3.26465E-018X_1_X_2_	0.1628	0.6141
Hue	+105.14579–1.05418X_1_ -0.24167X_2_ + 0.010643${\text{X}}_{1}^{2}$ + 2.53147E-003${\text{X}}_{2}^{2}$ + 2.46716E-017X_1_X_2_	0.0284∗	0.7822
Chroma	+12.26921 + 0.023154X_1_ -7.33503E-004X_2_	0.0635	0.4238

**Sorghum flour**			

L∗	+74.2564–4.61294E-003X_1_ – 0.062396X_2_ -8.02951E -0.0051E-005${\text{X}}_{1}^{2}$ + 7.18316E-004${\text{X}}_{2}^{2}$ -1.73611E -005X_1_X_2_	0.0258∗	0.7886
a∗	+2.82555–0.019699X_1_ +4.27083E-003X_2_ + 3.17925E-004${\text{X}}_{1}^{2}$ -5.9983E -005${\text{X}}_{2}^{2}$ -3.13214E-019X_1_X_2_	0.0004∗	0.9414
b∗	+10.69008 + 0.13042X_1_ +0.26073X_2_ +1.4041-003${\text{X}}_{1}^{2}$ -2.68338E-003${\text{X}}_{2}^{2}$ + 4.04769E-018X_1_X_2_	0.0352∗	0.7668
Hue	+79.36825 + 0.059708X_1_ -0.033004X_2_ -1.34983E-003${\text{X}}_{1}^{2}$ + 3.99306E-004${\text{X}}_{2}^{2}$ -4.34028E-006X_1_X_2_	0.0015∗	0.9106
chroma	+14.59497–0.017083X_1_ +3.85417E-003X_2_	0.0265∗	0.5162

∗Significant at p < 0.05, ∗∗Non-significant p > 0.05, X_1_- linear effect of malting, X_2_- linear effect of fermentation, X_1_X_2_- the interaction of malting and fermentation, ${\text{X}}_{1}^{2}$ – quadratic effect of malting; ${\text{X}}_{2}^{2}$ – quadratic effect of fermentation.

**Figure 1:**
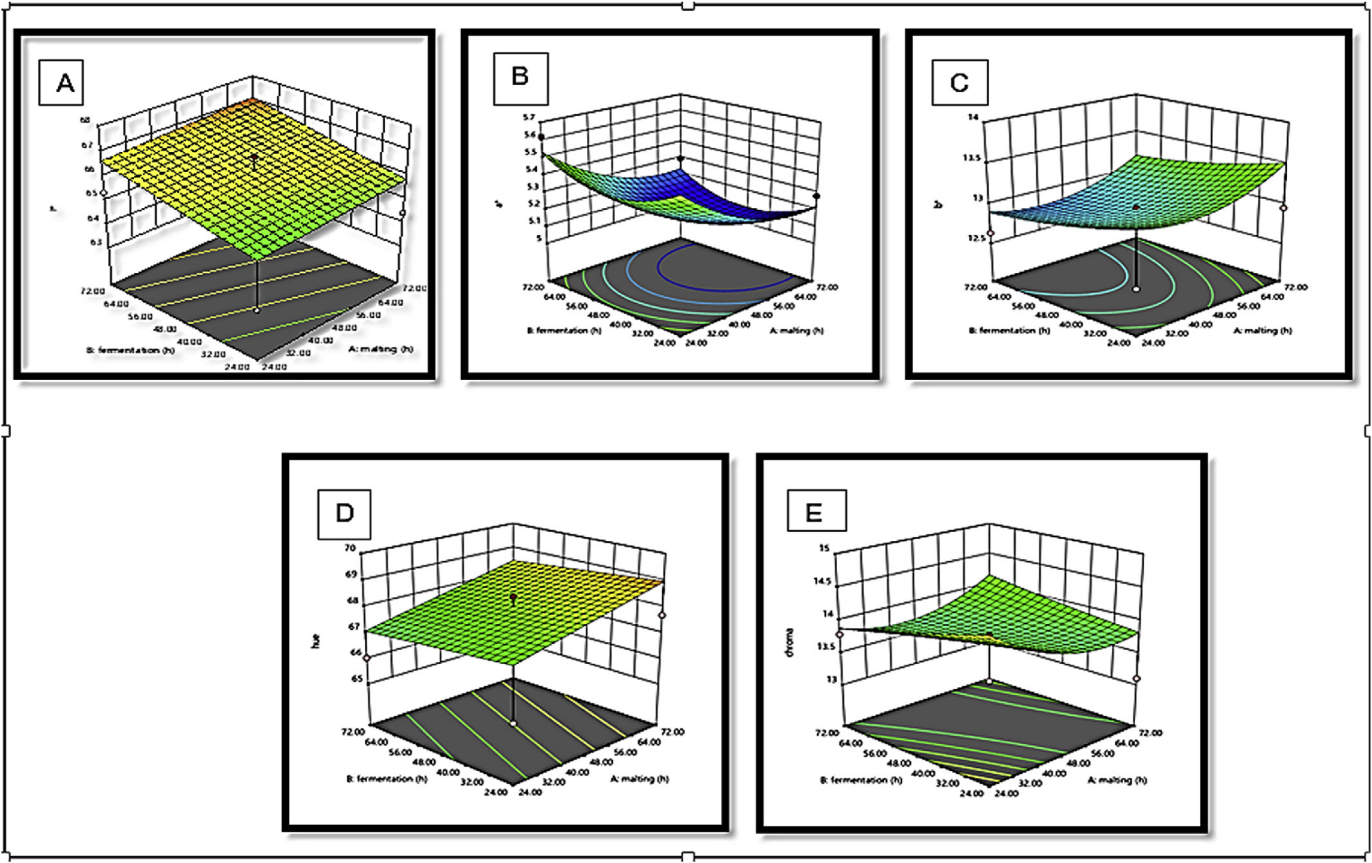
(A–E): Response surface plots for the effects of malting and fermentation: A = lightness (L∗), B = redness (a∗), C = yellowness (b∗), D = hue and E = chroma for *Agrigreen* pearl millet flour.

**Figure 2:**
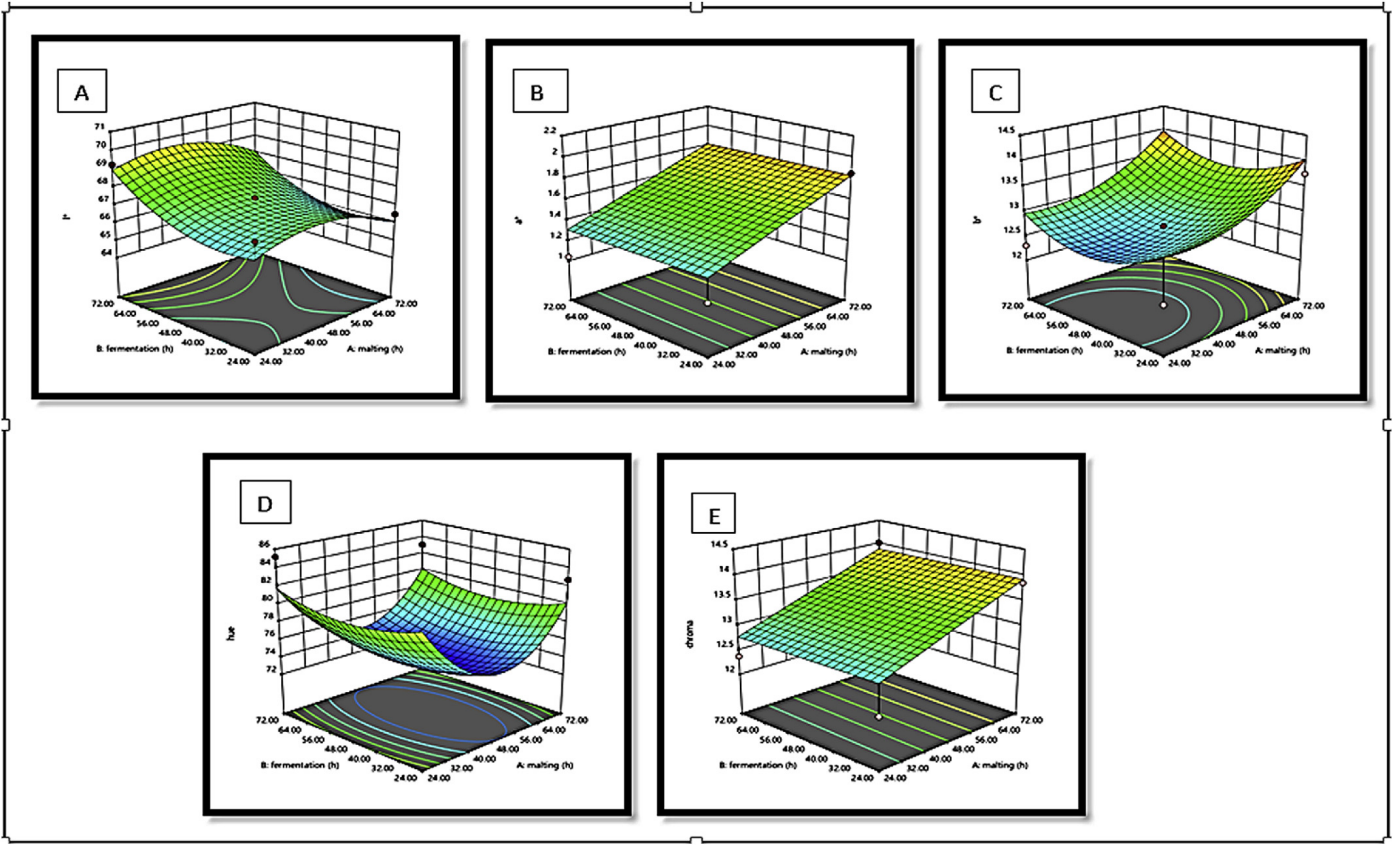
(A–E): Response surface plots for the effects of malting and fermentation: A = lightness (L∗), B = redness (a∗), C = yellowness (b∗), D = hue and E = chroma for *Babala* pearl millet flour.

**Figure 3:**
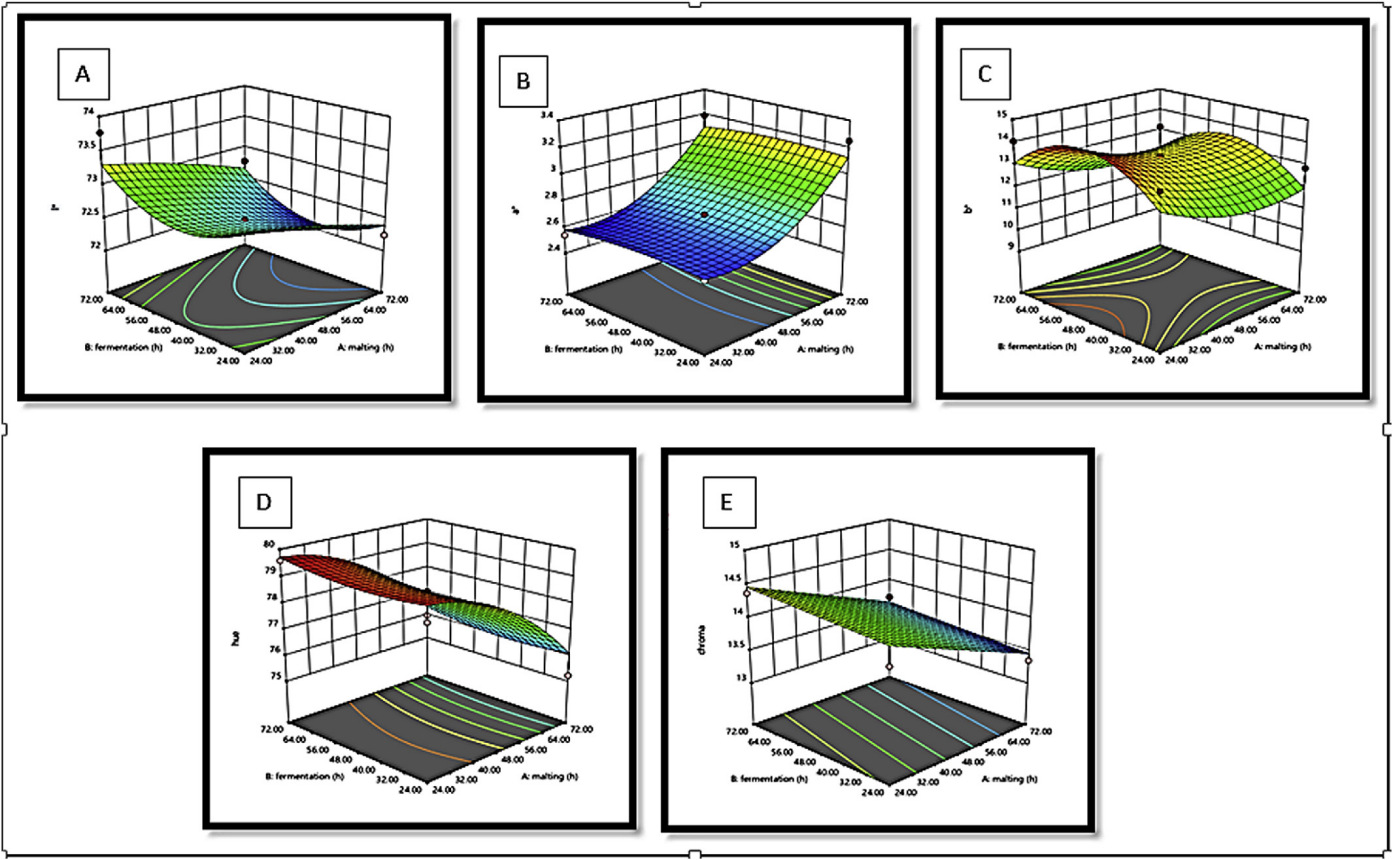
(A–E): Response surface plots for the effects of malting and fermentation: A = lightness (L∗), B = redness (a∗), C = yellowness (b∗), D = hue and E = chroma for sorghum.

The response surface plots of L∗, a∗ b∗, hue and chroma with a variation in malting and fermentation for *Agrigreen*, *Babala* cultivars of pearl millet and sorghum flours showed that *Agrigreen* flour exhibited an increase in L∗, b∗ and hue upon the increase in malting and fermentation time, while increment in a∗ and chroma were inversely proportional to the malting and fermentation time (Figure 1). In *Babala* flour, an increase in malting and fermented time increased L∗, a∗, b∗, hue and chroma (Figure 2). For sorghum flour, the decrease in malting time increased the level of lightness (L∗), while increment in fermentation time increased the level of the lightness (L∗) (Figure 3). The redness (a∗) was increased as the malting and the fermentation times increased. An increase in malting and fermentation time reduced the yellowness (b∗), while hue was reduced upon an increase in mating and fermentation time. The level of chroma was reduced as the malting time increase, while an increase in fermentation time increased the level of chroma. Omolola et al. (2015) and Akinoso and Adeyanju (2012) stated that response surface plots assist to envisage the shape of the response surface and provide valuable information about the fitness of the model. These differences in colour parameters upon different processing times could be attributed to the difference in flours of pearl millet cultivars and sorghum, chemical changes in the colour pigment during malting and fermentation time due to the oxidation of phenolic acids (Taylor and Duodu, 2015) and differential in species.

### 3.2 Effect of malting and fermentation time on thermal properties of pearl millet and sorghum flours

The effect of malting and fermentation time on thermal properties of *Agrigreen*, *Babala* and sorghum flours show that onset (*T*_*o*_ ⁰C), peak melting (*T*_*p*_ ⁰C), end completion (*T*_*c*_ ⁰C) gelatinisation range (***Δ****T*_*r*_ ⁰C) and gelatinisation enthalpy (J/g) were varied processing time (h). The *T*_*o*_ values of malted and fermented *Agrigreen* flour ranged from 88.44 to 98.33 °C (Table 7), malted and fermented *Babala* flour was 93.20–100.11 °C (Table 8) while malted and fermented sorghum flour had 77.11–98.27 °C (Table 9). The results indicate that 48 h malted and 14 h fermented *Babala* flour requires high temperatures to initiate gelatinisation at 100.11 °C compared to malted and fermented *Agrigreen* and sorghum flours that gelatinised at 98.33 °C and 98.27 °C respectively. The obtained results were similar to Jideani and Scott (2012) for the hydrated cooked pearl millet. The range of the *T*_*p*_ value of *Agrigreen* was 95.70–104.50 °C; *Babala* was 95.46–105.71 °C and sorghum was 87.57–104.83 °C. *Babala* flour exhibited high content of peak melting at 48 h mating and 14 h fermentation time. Though, *T*_*p*_ for the malted and fermented *Agrigreen*, *Babala* and sorghum flours were closer to each other, showing the exact point where starch granules present in the sample had broken into smaller units. The melting peak of the malted sorghum and pearl millet was higher than 65–70 °C of Sozer et al. (2007) and 74.4–76.15 °C reported by Ahmed et al. (2016), but closer to the result of Jideani and Scott (2012). The reason for high gelatinisation in the flour of the malted and fermented flour of pearl millet cultivars and sorghum flour could result from the high presence and the ratio of amylopectin and amylose present in the starch granules, which were unable to break down starch to sugars during malting and fermentation times either by an enzymatic process such α-amylase or lactic acid bacteria (LAB). Similarly, high gelatinisation of malted and fermented of pearl millet cultivars and sorghum flours could be credited to the type of species of cereals produced in a particular area. The *T*_*c*_ was 102.57–109.26 °C for *Agrigreen*, 105.25–110.03 °C for *Babala* and 102.66–111.14 °C for sorghum flour. Relating the malting and fermentation time of flours, the results showed that sorghum flour exhibited the highest values for end completion at 81.94 h malting and 48 h fermentation time. The values of end completion of malted and fermented pearl millet cultivars and sorghum flour are higher than those reported by Dangi et al. (2019) and Ahmed et al. (2016) for composite guar gum-pearl millet flour and sorghum starch respectively, but lower than the results reported by Jideani and Scott (2012) for the hydrated cooked pearl millet. The ***Δ****T*_*r*_ of *Agrigreen* flour was 8.02–14.13 °C, 10.47–14.13 °C for *Babala* and sorghum was 10.70–25.79 °C. From the results, it could be deduced that malted and fermented sorghum flour had the highest gelatinisation range 23.02 °C at 24 h mating and 24 h fermentation time than malted and fermented *Agrigreen* and *Babala* flours. Variations in the gelatinisation of processed flour could due to protein content, starch structures (in terms of crystal structure) (Yang et al., 2019; Kaur and Singh, 2005). The melting enthalpy of starch-lipid complexes and protein-starch interaction that inhibits the absorption of water in the granule to swell up and gelatinise (Liu et al., 2007). Jayakody et al. (2005) described that an increment in a starch-lipid complex formation reduces the degree of hydration in the amorphous area, thus causing the amount of thermal energy required to melting. Gelatinisation ranges (*ΔT*_*r*_) are correlated to the results of cooked pearl millet flour reported by Jideani and Scott (2012).

**Table 7:** Effect of processing time on thermal properties of *Agrigreen* pearl millet flour.

Runs	Malting (h)	Fermentation (h)	Onset (T_o_) ⁰C	Peak melting (T_p_) ⁰C	End completion (T_c_) ⁰C	Gelatinisation range ΔT_r_ = (T_c_-T_o_) °C	Enthalpy J/g
1	48.00	48.00	98.33 ± 1.40	104.50 ± 0.87	109.26 ± 1.32	10.93 ± 1.32	6.96 ± 1.93
2	24.00	24.00	91.94 ± 0.26	98.73 ± 0.21	104.78 ± 0.13	12.84 ± 0.20	5.95 ± 0.97
3	48.00	48.00	98.33 ± 1.40	104.50 ± 0.87	109.26 ± 1.32	10.93 ± 1.32	6.96 ± 1.93
4	48.00	48.00	98.33 ± 1.40	104.50 ± 0.87	109.26 ± 1.32	10.93 ± 1.32	6.96 ± 1.93
5	24.00	72.00	92.80 ± 0.47	100.09 ± 0.13	106.47 ± 0.53	13.67 ± 0.41	7.68 ± 1.48
6	48.00	48.00	98.33 ± 1.40	104.50 ± 0.87	109.26 ± 1.32	10.93 ± 1.32	6.96 ± 1.93
7	48.00	48.00	98.33 ± 1.40	104.50 ± 0.87	109.26 ± 1.32	10.93 ± 1.32	6.96 ± 1.93
8	72.00	72.00	97.63 ± 0.63	103.62 ± 0.13	109.50 ± 0.25	11.87 ± 0.47	7.35 ± 1.96
9	14.06	48.00	92.77 ± 0.26	98.71 ± 0.61	104.53 ± 0.01	11.76 ± 0.17	6.12 ± 0.11
10	48.00	14.06	88.44 ± 2.42	95.70 ± 1.42	102.57 ± 2.86	14.13 ± 1.92	6.08 ± 0.69
11	81.94	48.00	98.04 ± 1.80	104.26 ± 1.22	109.26 ± 1.32	10.48 ± 1.67	8.61 ± 0.41
12	72.00	24.00	96.78 ± 1.42	102.27 ± 0.21	107.80 ± 0.85	11.02 ± 1.17	5.61 ± 0.49
13	48.00	81.94	94.54 ± 1.05	101.82 ± 1.08	102.56 ± 2.84	8.02 ± 1.86	7.52 ± 2.73

**Table 8:** Effect of processing time on thermal properties of *Babala* pearl millet flour.

Runs	Malting (h)	Fermentation (h)	Onset (T_o_) ⁰C	Peak melting (T_p_) ⁰C	End completion (T_c_) ⁰C	Gelatinisation range ΔT_r_ = (T_c_-T_o_) °C	Enthalpy (J/g)
1	48.00	48.00	98.88 ± 1.26	104.81 ± 0.23	110.03 ± 2.36	11.23 ± 1.76	14.65 ± 1.66
2	24.00	24.00	88.23 ± 1.20	95.46 ± 1.30	102.36 ± 1.31	14.13 ± 1.36	6.11 ± 1.43
3	48.00	48.00	98.88 ± 1.26	104.81 ± 0.23	110.03 ± 2.36	11.23 ± 1.76	14.65 ± 1.66
4	48.00	48.00	98.88 ± 1.26	104.81 ± 0.23	110.03 ± 2.36	11.23 ± 1.76	14.65 ± 1.66
5	24.00	72.00	93.20 ± 1.20	100.26 ± 1.09	105.24 ± 1.39	12.04 ± 1.34	20.93 ± 0.38
6	48.00	48.00	98.88 ± 1.26	104.81 ± 0.23	110.03 ± 2.36	11.23 ± 1.76	14.65 ± 1.66
7	48.00	48.00	98.88 ± 1.26	104.81 ± 0.23	110.03 ± 2.36	11.23 ± 1.76	14.65 ± 1.66
8	72.00	72.00	98.16 ± 1.18	104.38 ± 1.81	109.40 ± 2.50	11.24 ± 1.81	21.62 ± 0.40
9	14.06	48.00	95.85 ± 0.56	103.26 ± 1.41	109.42 ± 0.22	13.57 ± 0.44	17.28 ± 1.94
10	48.00	14.06	100.11 ± 0.1	105.71 ± 0.50	110.58 ± 1.15	10.47 ± 0.59	5.72 ± 1.03
11	81.94	48.00	89.94 ± 0.89	97.16 ± 0.04	103.56 ± 0.51	13.62 ± 0.67	14.99 ± 0.19
12	72.00	24.00	93.22 ± 1.19	99.74 ± 0.25	106.52 ± 0.42	13.30 ± 0.78	6.80 ± 1.44
13	48.00	81.94	93.57 ± 1.76	99.96 ± 1.37	106.24 ± 0.01	12.67 ± 0.98	8.57 ± 0.06

**Table 9:** Effect of processing time on thermal properties of sorghum flour.

Runs	Malting (h)	Fermentation (h)	Onset (T_o_) °C	Peak melting (T_p_) °C	End completion (T_c_) °C	Gelatinisation range ΔT_r_ = (T_c_-T_o_) °C	Enthalpy (J/g)
1	48.00	48.00	92.51 ± 1.01	98.40 ± 1.96	104.57 ± 0.33	12.06 ± 0.79	17.74 ± 0.83
2	24.00	24.00	81.23 ± 0.20	90.54 ± 0.46	104.25 ± 0.37	23.02 ± 0.29	21.36 ± 1.11
3	48.00	48.00	92.51 ± 1.01	98.40 ± 1.96	104.57 ± 0.33	12.06 ± 0.79	17.74 ± 0.83
4	48.00	48.00	92.51 ± 1.01	98.40 ± 1.96	104.57 ± 0.33	12.06 ± 0.79	17.74 ± 0.83
5	24.00	72.00	77.11 ± 0.87	87.57 ± 1.25	102.90 ± 1.47	25.79 ± 0.89	16.71 ± 1.54
6	48.00	48.00	92.51 ± 1.01	98.40 ± 1.96	104.57 ± 0.33	12.06 ± 0.79	17.74 ± 0.83
7	48.00	48.00	92.51 ± 1.01	98.40 ± 1.96	104.57 ± 0.33	12.06 ± 0.79	17.74 ± 0.83
8	72.00	72.00	91.20 ± 1.96	99.70 ± 1.09	107.59 ± 1.84	16.39 ± 1.86	15.38 ± 1.42
9	14.06	48.00	92.03 ± 1.71	99.09 ± 1.99	106.12 ± 0.92	14.09 ± 1.28	19.92 ± 0.76
10	48.00	14.06	94.28 ± 1.50	99.71 ± 0.82	104.98 ± 0.91	10.70 ± 1.19	13.27 ± 0.51
11	81.94	48.00	98.27 ± 1.89	104.83 ± 1.86	111.14 ± 0.03	12.87 ± 0.89	19.54 ± 0.29
12	72.00	24.00	95.31 ± 0.78	102.68 ± 1.30	108.94 ± 1.74	13.63 ± 1.18	20.03 ± 1.99
13	48.00	81.94	87.19 ± 1.47	94.71 ± 1.75	102.66 ± 1.63	15.47 ± 1.47	14.36 ± 0.05

The enthalpy of malted and fermented *Agrigreen* was in the range 5.95–8.61 J/g, 5.72–21.62 J/g for *Babala* and 13.27–20.03 J/g for sorghum flour. *Babala* relatively recorded high enthalpy at 72 h malting and 72 h fermentation time than other *Agrigeen* and sorghum flour. This means that malted and fermented sorghum flour exhibited the highest energy to melt starch granules. The enthalpy results of the malted and fermented flours were within the reported range for sorghum starch by Ahmed et al. (2016), but higher than guar-pearl millet results reported by Dangi et al. (2019) but lower than the reported results of Jideani and Scott (2012). Variation of end completion and enthalpy in malted and fermented *Agrigree*n, *Babala* and sorghum flours were attributed to starch gelatinisation, which might be hindered by the presence of protein, melting enthalpy, starch structure and lipid-starch complexes (Liu et al., 2007; Chung et al., 2008). Anyasi et al. (2017) reported that gelatinisation of flour brings disruption or collapse of molecular granules with irresistible variations in properties such as granular swelling, native crystallite melting, loss of birefringence and starch solubilisation. Considering the obtained results of the processed flour, it was evident that processing times such as malting and fermentation influenced the thermal properties. These variations in thermal properties may be a result of starch granules containing amylose and amylopectin which was difficult to be broken into smaller units during processing time, thereby requires high energy to gelatinise. These unbroken granules could be attributed to starch-protein complexes (Liu et al., 2007).

The analysis of variance (ANOVA) of the effect of model parameters on the thermal properties of *Agrigreen*, *Babala* and sorghum is shown in Table 10. The ANOVA table indicated linear interactions of malting (X_1_) and fermentation (X_2_) time, interaction effects of malting and fermentation time (X_1_X_2_), reduced quadratics effects on malting and fermentation time (${\text{X}}_{1}^{2}$${\text{X}}_{2}^{2}$) and cubic effects on malting and fermentation time (${\text{X}}_{1}^{3}$ ${\text{X}}_{2}^{3}$) on thermal properties of the flour at significant value p < 0.05 for the onset, melting point, completion time, gelatinisation range and enthalpy. Linear interaction effects (X_1_ and X_2_) were mostly exhibited in *Agrigreen*, *Babala* and sorghum flours upon malting (X_1_) and fermentation (X_2_) time for onset, peak melting, end completion, gelatinisation range and enthalpy. Quadratic effect (${\text{X}}_{1}^{2}$ and ${\text{X}}_{2}^{2}$) and interaction effect (X_1_ X_2_) were noticed in onset, peak melting, end completion, gelatinisation range and enthalpy of *Agrigreen*, *Babala* and sorghum flours during malting and fermentation time, while cubic effects (${\text{X}}_{1}^{3}$ ${\text{X}}_{2}^{3}$) were displayed in end completion and enthalpy of *Babala* and sorghum flours during processing. It could be inferred that malting and fermentation processing time affected the thermal properties of pearl millet cultivars and sorghum flours.

**Table 10:** ANOVA results of the effect of model parameters on thermal properties of *Agrigreen, Babala* pearl millet and sorghum flours.

Source	Onset (°C)		Peak melting (°C)		End completion (°C)		Gelatinisation range (°C)		Enthalpy (J/g)	
	F value	P value	F value	P value	F value	P value	F value	P value	F value	P value
***Agrigreen* flour**										
Model	22.04	0.0004∗	20.21	0.0005∗	7.97	0.0083∗	2.59	0.1240∗∗	7.57	0.0100∗
X_1_	30.46	0.0009∗	28.98	0.0010∗	10.85	0.0132∗	1.96	0.1917∗∗	3.30	0.0993∗
X_2_	13.92	0.0073∗	16.82	0.0046∗	0.76	0.4117∗∗	3.22	0.1029∗∗	11.84	0.0064∗
${\text{X}}_{1}^{2}$	8.70	0.0214∗	11.21	0.0123∗	1.26	0.2997∗∗				
${\text{X}}_{2}^{2}$	61.93	0.0001∗	49.22	0.0002∗	28.04	0.0011∗				
X_1_X_2_	0.12	0.7382∗∗	2.604e-005	0.9961∗∗	1.337e-005	0.9972∗∗				

***Babala* flour**										

Model	1.32	0.3547∗∗	1.02	0.4724∗∗	13.4	0.0060∗	2.12	0.1770∗∗	4.08	0.0507∗
X_1_	0.024	0.8818∗∗	5.641e-004	0.9817∗∗	31.51	0.0025∗	0.31	0.5978∗∗	1.12	0.3147∗∗
X_2_	4.0976e-003	0.9508∗∗	0.019	0.8948∗∗	15.81	0.0106∗	0.14	0.7237∗∗	7.03	0.0242∗
${\text{X}}_{1}^{2}$	6.06	0.0043∗	4.42	0.0736∗∗	32.99	0.0022∗	10.17	0.0153∗		
${\text{X}}_{2}^{2}$	1.08	0.3340∗∗	1.6	0.3171∗∗	10.20	0.0242∗	0.26	0.6243∗∗		
X_1_X_2_	1.688e-005	0.9968∗∗	5.602e-004	0.9817∗∗	0.000	1.000∗∗	2.260e-004	0.9884∗∗		
${\text{X}}_{1}^{3}$					34.97	0.0020∗				
${\text{X}}_{2}^{3}$					17.95	0.0082∗				

**Sorghum flour**										

Model	5.79	0.0214∗	7.73	0.0093∗	109.17	<0.0001∗	1.57	0.2834∗∗	8.28	0.0167∗
X_1_	9.31	0.0122∗	13.31	0.0045∗	270.51	<0.0001∗	3.11	0.1214∗∗	1.12	0.3383∗∗
X_2_	2.27	0.1630∗∗	2.15	0.1732∗∗	35.63	0.0006∗	1.14	0.3215∗∗	19.87	0.0067∗
${\text{X}}_{1}^{2}$					211.61	<0.0001∗	2.19	0.1823∗∗	13.25	0.0149∗
${\text{X}}_{2}^{2}$					11.29	0.0121∗	1.88	0.2130∗∗	16.65	0.0095∗
X_1_X_2_					0.000	1.000∗∗	1.476e-006	0.9991∗∗	0.000	1.000∗∗
${\text{X}}_{1}^{3}$									0.55	0.491∗∗
${\text{X}}_{2}^{3}$									14.39	0.0127∗

∗Significant at p < 0.05, ∗∗Non-significant at p > 0.05, X_1_- linear effect of malting, X_2_- linear effect of fermentation, X_1_X_2_- the interaction of malting and fermentation, ${\text{X}}_{1}^{2}$ – quadratic effect of malting; ${\text{X}}_{2}^{2}$ – quadratic effect of fermentation, ${\text{X}}_{1}^{3}$ - cubic effect of malting, ${\text{X}}_{2}^{3}$ - cubic effect of fermentation.

The regression models of thermal properties and processing times for *Agrigreen, Babala* and sorghum flours indicated the effects of relationship upon processing (Table 11). Models showed linear, quadratic and interactions upon processing techniques such as malting and fermentation on the thermal properties such as onset, peak melting, end completion, gelatinisation range and enthalpy. Virtually all the treated flours showed positive intercepts, indicating that the relationship between the malting and fermentation time are positively related. The degree of their positive values on the processing times differs based on their contribution to each processing method. For malted and fermented *Agrigreen* flour, malting (X_1_) had the utmost linear effect on the onset, peak melting and enthalpy, quadratic effects were high on the onset and end completion. Fermentation (X_2_) of *Agrigreen* flour had a most linear effect on the onset, peak melting, end completion and enthalpy, the quadratic effect was not noticed; and interaction effects on the onset and end completion were noticed high. *Babala* flour showed that malting (X_1_) had the most effect on the linear onset, peak melting and end completion without any quadratic effect, while fermentation (X_2_) had most linear effects on the onset, peak melting, end completion and enthalpy. Quadratic and cubic effects were not noticed in *Babala* flour. While the interaction effects on *Babala* flour (X_1_X_2_) were most high on the onset and end of completion. For sorghum flour, malting (X_1_) had the most effect on the onset, quadratic effect on end completion and cubic on enthalpy while fermentation exhibited the most linear effects on end completion and enthalpy. Quadratic cubic effects were noticed on enthalpy while the interaction effect was most high on end completion and enthalpy. The effects of the processed flour showed relatively high R^2^ values. Having low R^2^ values in gelatinisation range of *Agrigreen* flour, peak melting and enthalpy of *Babala* flour at 0.441, 0.421 and 0.449 respectively does not mean that the models are insignificant. Indication of low r^2^ values could be a result of having many data points that show little explanation between the dependent and independent variables. Though gealtinisation range of *Agrigreen* flour in was noticed high at 13.67 °C for 24 h malted and 72 h fermentation time (Table 7). While peak melting ⁰C and enthalpy (J/g) of *Babala* flour was 105.21 °C at 48 h malted and 14.06 h fermentation time and 21.62 (J/g) at 72 h malted and 72 h fermentation time respectively (Table 8). A lack of a t-test of the models was non- significant at p > 0.05. A non-significant lack of t-test is good as this strengthens the fitness of the models coupled with a high coefficient of variation (R^2^). This guarantees good fitness of the model when applied. The coefficients of the model's parameters show the degree and significance of each model factor with regards to their effects on the response variables, that is; the higher the coefficient of a model parameter, the greater the significance of the parameter (Jideani and Scott, 2012; Omolola et al., 2015).

**Table 11:** Regression models relating thermal properties response and independent variables for *Agrigreen*, *Babala* pearl millet and sorghum flours.

Response variables	Models	Residual fit at p value	R^2^
***Agrigreen* flour**			
Onset (°C)	+74.975 + 0.2657 X_1_ +0.5644 X_2_ -2.0377E-003 ${\text{X}}_{1}^{2}$ -5.436E-003 ${\text{X}}_{2}^{2}$ + 3.168E-004 X_1_ X_2_	0.0004∗	0.940
Peak melting (°C)	+82.519 + 0.285 X_1_ +0.4938 X_2_ – 2.1593E-003 X_1_-4.5247E-003 X_2_ – 4.34028E-006 X_1_ X_2_	0.0005∗	0.935
End completion (°C)	+91.9362 + 0.1629 X_1_ + 0.47498 X_2_ – 1.008E-003 ${\text{X}}_{1}^{2}$ – 4.7667E-003 ${\text{X}}_{2}^{2}$ + 4.34028E-006 X_1_ X_2._	0.0083∗	0.851
Gelatinisation range (°C)	+14.51622–0.028282 X_1_ -0.036254 X_2_	0.1240	0.441
Enthalpy (J/g)	+4.79485 + 0.015146 X_1_ + 0.0286 X_2_	0.0100∗	0.602

***Babala* flour**			

Onset (°C)	+78.706 + 0.57704 X_1_ + 0.2434 X_2_ – 5.91797E-003${\text{X}}_{1}^{2}$ – 2.2934E-003 ${\text{X}}_{2}^{2}$ – 1.30208E-005 X_1_ X_2_	0.3547∗∗	0.486
Peak melting (°C)	+88.1046 + 0.4506 X_1_ +0.2398 X_2_ - 4.6712E-003 ${\text{X}}_{1}^{2}$ -6.944E-005 X_1_ X_2._	0.4724∗∗	0.421
End completion (°C)	+110.03 + 6.23 X_1_ + 4.41 X_2_ - 2.16 ${\text{X}}_{1}^{2}$ - 1.20 ${\text{X}}_{2}^{2}$ + 0.00 X_1_ X_2_ - 4 .15 ${\text{X}}_{1}^{3}$ -2.97 ${\text{X}}_{2}^{3}$	0.0060∗	0.949
Gelatinisation range (°C)	+17.50950–0.20979X_1_ - 0.038327X_2_ +2.09418E-003${\text{X}}_{1}^{2}$ + 3.36372E-004 ${\text{X}}_{2}^{2}$ + 1.30208E-005X_1_ X_2_	0.177∗∗	0.603
Enthalpy (J/g)	+7.7949–0.070005 X_1_ + 0.17537 X_2_	0.0507∗∗	0.449

**Sorghum flour**			

Onset (°C)	+86.021 + 0.1927 X_1_ -0.0951 X_2_	0.0214∗	0.537
Peak melting (°C)	+92.915 + 0.1687 X_1_ – 0.0678 X_2_	0.0093∗	0.607
End completion (°C)	+107.955–0.2398 X_1_ + 0.0441 X_2_ +3.3919E-003 ${\text{X}}_{1}^{2}$ - 7.834E-004 ${\text{X}}_{2}^{2}$ + 9.0109E-017 X_1_ X_2_	<0.0001∗	0.987
Gelatinisation range (°C)	+31.86453–0.49164 X_1_ -0.29137 X_2_ +4.01042E-003 ${\text{X}}_{1}^{2}$ + 3.71094E-003 ${\text{X}}_{2}^{2}$ - 4.34028E-006 X_1_ X_2_	0.2834	0.529
Enthalpy (J/g)	+17.74–1.20 X_1_ -5.04 X_2_ +1.39 ${\text{X}}_{1}^{2}$ – 1.56 ${\text{X}}_{2}^{2}$ + 0.000 X_1_ X_2_ + 0.53 ${\text{X}}_{1}^{3}$ + 2.71 ${\text{X}}_{2}^{3}$	0.0167∗	0.921

∗Significant at p < 0.05, ∗∗non-significant p > 0.05, X_1_- linear effect of malting, X_2_- linear effect of fermentation, X_1_X_2_- the interaction of malting and fermentation, ${\text{X}}_{1}^{2}$ – quadratic effect of malting; ${\text{X}}_{2}^{2}$ – quadratic effect of fermentation, ${\text{X}}_{1}^{3}$ - cubic effect of malting, ${\text{X}}_{2}^{3}$ - cubic effect of fermentation.

**Figure 4:**
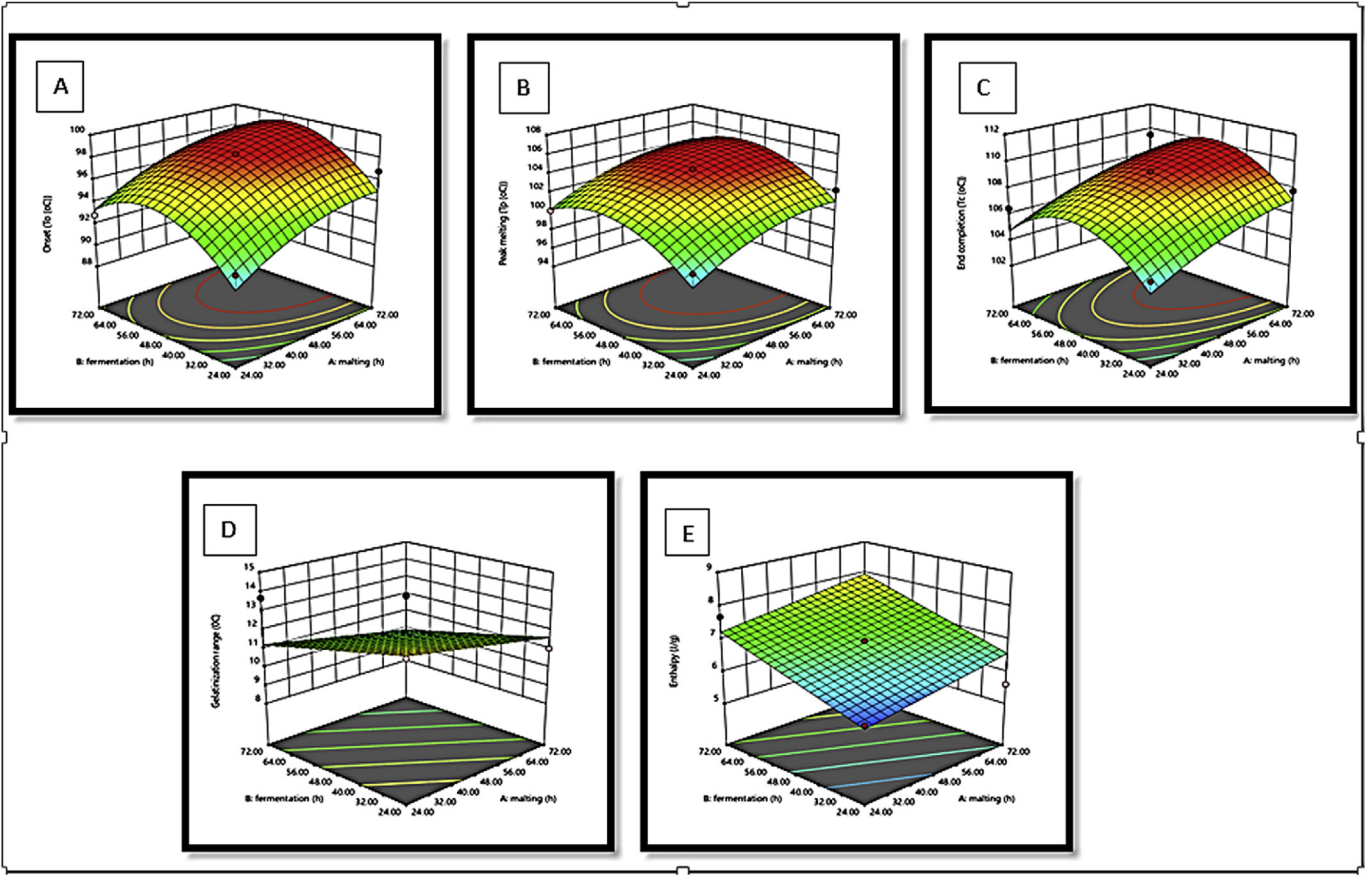
(A–E). Response surface plots for the effect of malting and fermentation time (h) on: A = onset, B = peak melting, C = end completion, D = gelatinisation range and E = enthalpy of *Agrigreen* pearl millet flour.

**Figure 5:**
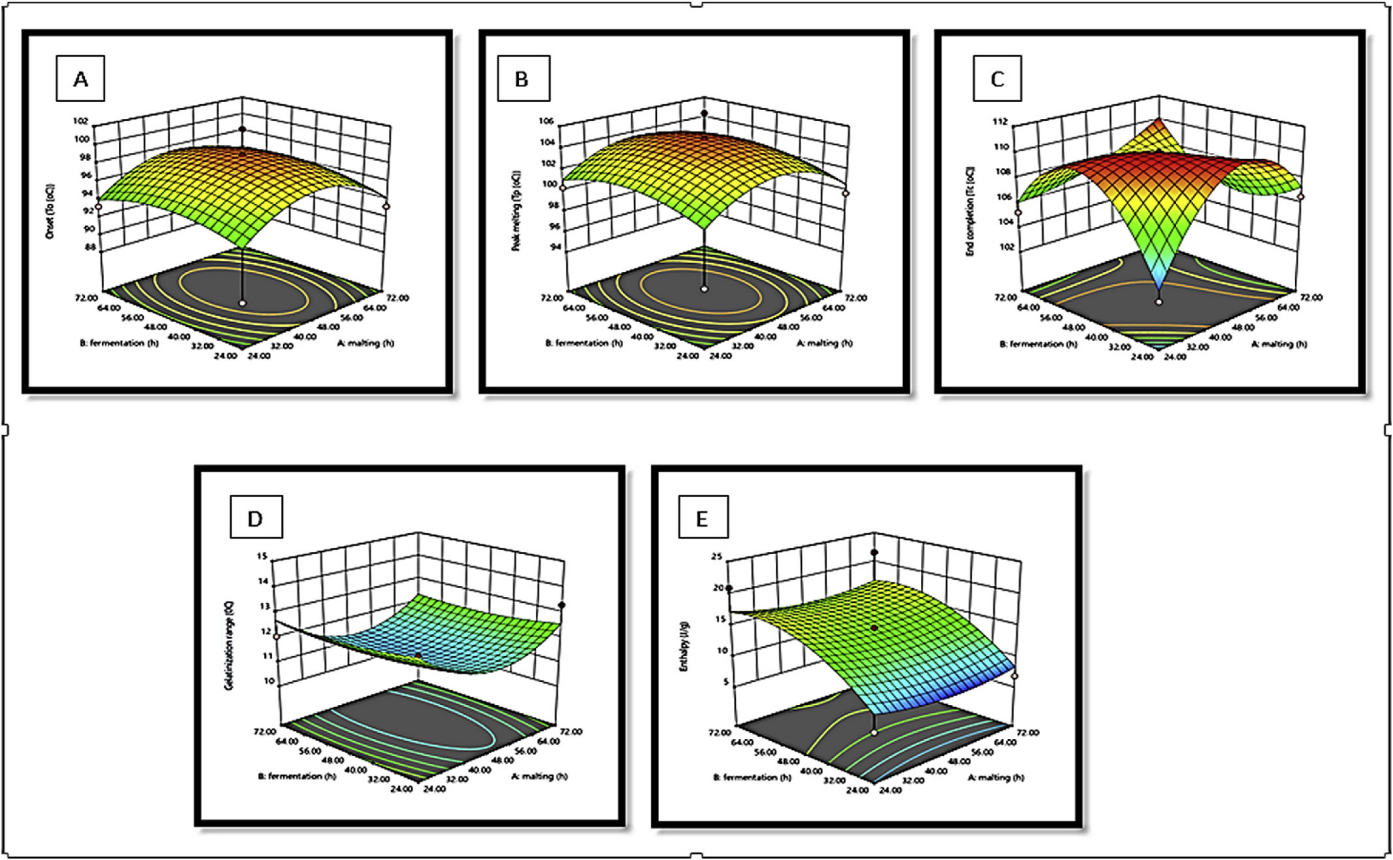
(A–E). Response surface plots for the effect of malting and fermentation time (h) on: A = onset, B = peak melting, C = end completion, D = gelatinisation range and E = enthalpy of *Babala* pearl millet flour.

**Figure 6:**
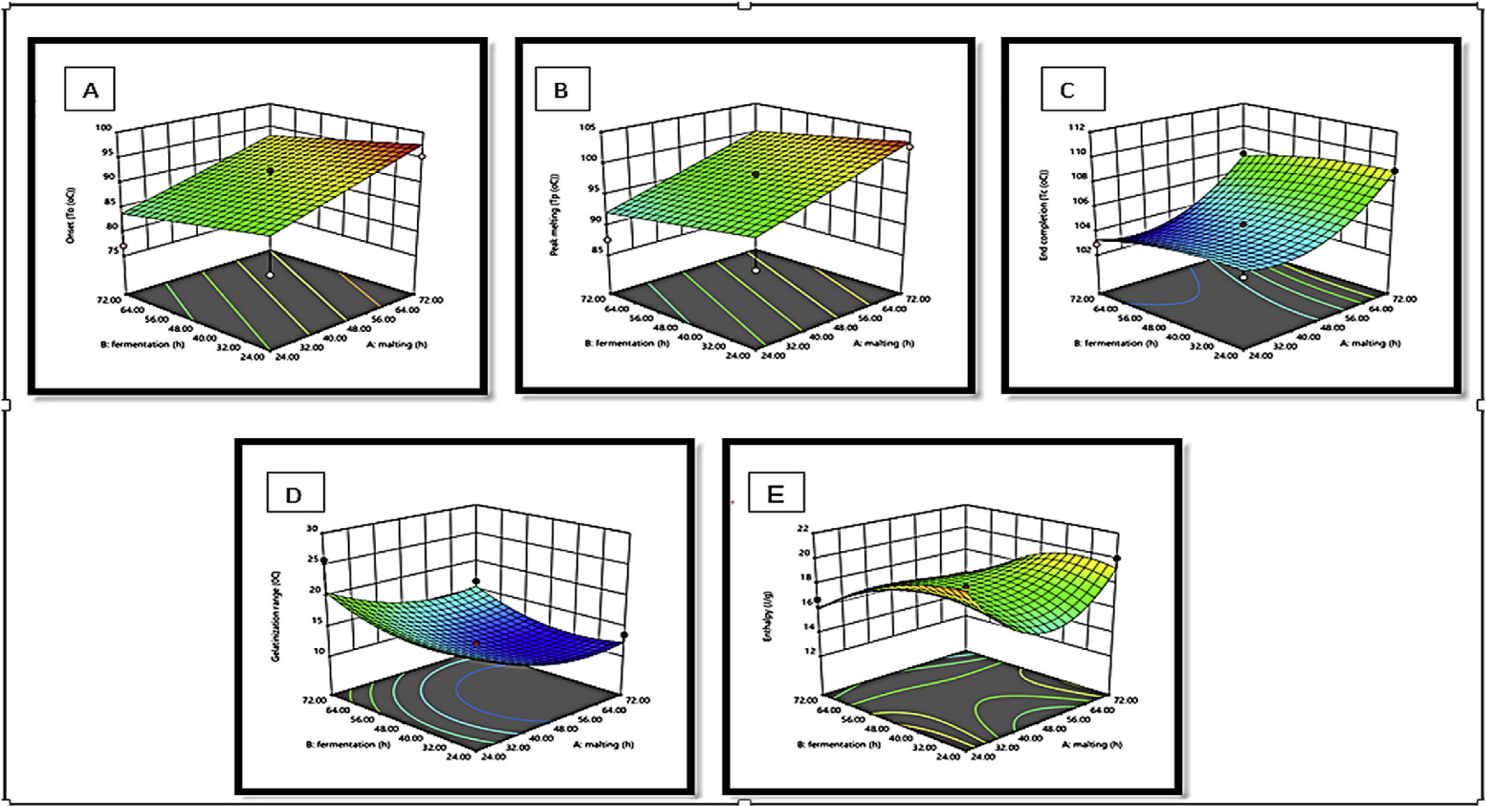
(A–E). Response surface plots for the effect of malting and fermentation time (h) on: A = onset, B = peak melting, C = end completion, D = gelatinisation range and E = enthalpy of sorghum flour.

The response surface plots showed the variability of onset, peak melting, end completion, gelatinisation range and enthalpy on malting and fermentation processing time (h) of *Agrigreen*, *Babala* and sorghum flours (Figures 4, 5, and 6). The response surface plots help to visualise the shapes of the contours and give useful evidence about the model's fitness (Omolola et al., 2015; Akinoso and Adeyanju, 2012). It could be deduced from the figures that there were differences in the shapes of the response surface plots obtained for *Agrigreen*, *Babala* and sorghum flours upon malting and fermentation processing conditions. These changes in response surface plots can be credited to the influence of malting and fermentation on pearl millet (*Agrigreen* and *Babala*) and sorghum flours under processing conditions. For malted and fermented *Agrigreen* flour, it could be inferred that an increase in malting and fermentation time increased the onset, peak melting, end completion and enthalpy, while malting and fermentation times were inversely for the gelatinisation range. Similarly, malted and fermented *Babala* flour showed an increase in onset, peak malting and end completion as the malting and fermentation time increased but with inverse proportional to the gelatinisation range and enthalpy. Malted and fermented sorghum flour exhibited an increase in onset, peak malting, and end completion upon an increase in malting and fermentation time. But the inverse relationship was noticed on the gelatinisation range and enthalpy during malting and fermentation time.

### 3.3 X-ray diffraction analysis of flour

**Figure 7:**
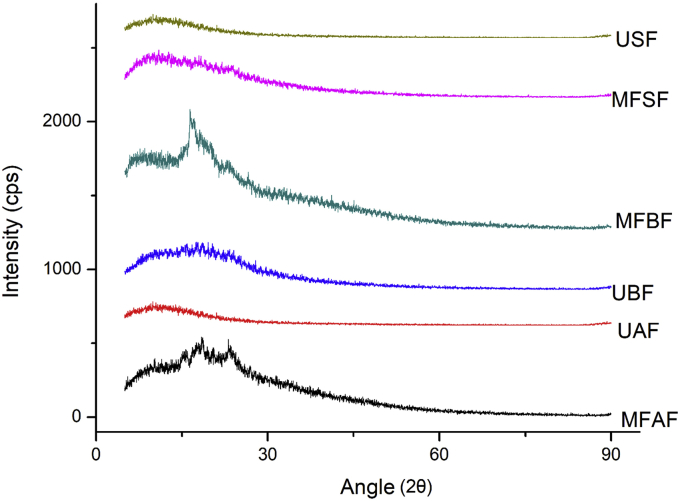
Level of crystallinity of processed and unprocessed flour of pearl millet cultivars and sorghum. (MFAF- malted and fermented Agrigreen flour; UAF- Unprocessed *Agrigreen* flour; MFBF- Malted and fermented *Babala* flour; UBF- Unprocessed *Babala* flour; MFSF- Malted and fermented sorghum flour; USF- Unprocessed sorghum flour).

The x-ray diffractograms of the malted and fermented flour at the optimum point and unprocessed flour of malted and fermented *Agrigreen* flour (MFAF), unprocessed *Agrigree*n flour (UAF), malted and fermented *Babala* flour (MFBF), unprocessed *Babala* flour (UBF), malted and fermented sorghum flour (MFSF), and unprocessed sorghum flour (USF) of *Agrigreen*, *Babala*, and sorghum flours are presented in Figure 7. It was evident that all the processed and unprocessed flour samples showed different diffraction patterns. The exhibited peaks’ intensities are relative to the quantity of arranged semi-crystalline structure and the differences in electron density between crystalline and amorphous lamellae (Hamley, 2003). The pointed peaks in the flour are correlated to the level of the crystallinity, while the diffused peaks quantified the amorphous region of the flour. All the samples (MFAF, UAF, MFBF, UBF, MFSF, and USF) demonstrated an A-type XRD pattern. MFAF had A-type diffraction form with main reflections at 2θ of 10.3°, 18.4°, and 26.9°, while UAF exhibited a diffuse peak at 9.9°. Related peaks were equally noticed for MFBF at 2θ for 12.8°, 16.6°, and 22.9°, while UBF had peaks at 17.7° and 19.9°. Flour MFSF and USF also showed diffraction patterns 2θ at 10.1°, 17.9°, 20.9°, and 9.2°, 13.9°, 21.1° respectively. Processed flour MFAF, MFBF and MFSF showed sharp peaks, this indicates the presence of crystallinity in the flour, while the unprocessed flour UAF, UBF and USF exhibited low and diffused peaks that signify amorphous region. From the diffractograms, it could be deduced that processing such as malting and fermentation had contributed to the breakdown of the starch granules which influenced the crystallinity of the flour. The main crystalline changes in diffraction arrangement specified the influence of malting and fermentation on amylose organisation, where processing remove amylopectin based ordered structure and generate amylose base order structure (Claver et al., 2010). A decrease in the intensities of the peaks may due to crystallite disruption or reorientation of the double helices creating the crystalline array due to the heat treatment (during drying) that gelatinises its starch (Dharmaraj et al., 2014). The A-typical arrangements of the diffraction patterns with high peaks were noticed in the processed and unprocessed flours of *Agrigreen*, *Babala* and sorghum were similar to other cereals reported by Kamble et al. (2019), Yang et al. (2019) and Amadou et al. (2014).

### 3.4 Fourier transform infrared (FTIR) spectroscopy of flour

**Figure 8:**
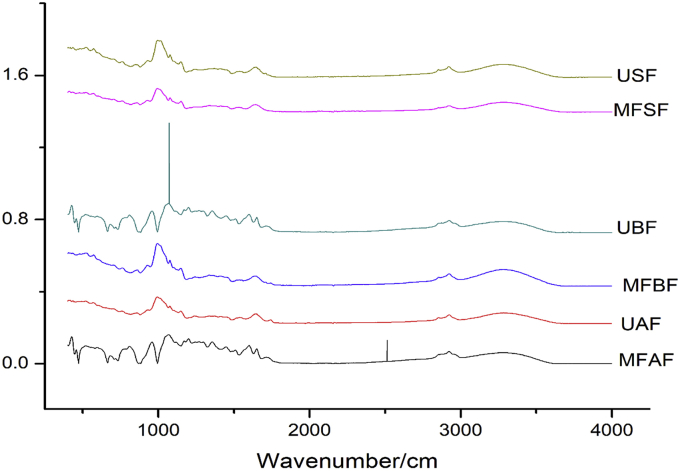
Spectra of functional groups of processed and unprocessed of pearl millet cultivars and sorghum flour (MFAF- malted and fermented *Agrigreen* flour; UAF- Unprocessed *Agrigreen* flour; MFBF- Malted and fermented *Babala* flour; UBF - Unprocessed *Babala* flour; MFSF- Malted and fermented sorghum flour; USF- Unprocessed sorghum flour).

FTIR spectroscopy for the functional groups of malted-fermented and unprocessed flour of pearl millet cultivars and sorghum is presented in Figure 8. The infrared (IR) spectra of the malted and fermented and unprocessed of pearl millet cultivars and sorghum flours showed different peaks with variations in intensity. The peaks of malted and fermented pearl millet and sorghum flour were in the range of 2999–3030 cm^−1^. Variations in the peaks could be credited to O–H bond stretching. Processed and unprocessed flours showed O–H absorption peaks from 3010 to 3030 cm^−1^ for MFAF and UAF, 3016 to 3003 cm^−1^ for MFBF and UBF and 3003 to 2999 cm^−1^ for MFSF and USF respectively. These changes in peaks could be related to increasing in the functional, better lipophilic and hydrophilic properties and the extent of inter and intramolecular bond of the MFAF, MFBF and MFSF flour sample (Sun et al., 2014). The results of Kamble et al. (2019) are in close range of peaks for the pasta produced from sorghum-finger millet with gluten. An increase in the wide range of MFAF, MFBF and MFSF samples spectrum could be attributed to the presence of alcohol, which was formed during fermentation. The asymmetrical stretching of the C–H band for the processed and unprocessed flours was in the range of 3655–3593 cm^−1^ while the vibrational peaks of the processed and unprocessed varied from 1540 to 1526 cm^−1^ The change in the vibrational peaks was due to the strongly bond found in the moisture of the flour. The carbonyl stretch bond of MFAF and UAF ranged from 1683 to 1679 cm^−1^, while 1672-1629 cm^−1^ were noticed in MFBF and UBF, and range 1683 and 1676 cm^−1^ were observed for MFSF and USF flour. Considering Figure 8, malting and fermentation time had reduced the carbonyl peaks due to total breakdown of lipid present in the flour (Correia et al., 2008). The C–O and aliphatic C–N stretching bonds of the processed and unprocessed flour ranged from 1153-1142 cm^−1^, while the N–H band was in the range 1683–1629 cm^−1^. The reduction in the strength of the band from 1683 to 1679 cm^−1^ in the MFAF and UAF, 1672 to 1629 cm^−1^ for MFBF and UBF, and 1683 to 1676 cm^−1^ for MFSF and USF flour reflect the changes in the level of crystallinity of the flour, due to the variation in the amylose and amylopectin. The obtained IR result is related to the results reported for maize and sorghum (Duodu et al., 2001; Correia et al., 2008; Adebiyi et al., 2016).

## 4 Conclusion

Malting and fermentation processing time influence the colour, thermal properties, crystallinity level and the functional groups of sorghum, *Agrigreen* and *Babala* pearl millet cultivars flours. There was a significant difference at p < 0.05 in the colour and thermal properties of malted and fermented flour of sorghum, *Agrigreen* and *Babala* pearl millet flour. Changes in the colour such as L∗, a∗, b∗, hue and chroma of malted and fermented flour of sorghum, *Agrigreen* and *Babala* millet flours could be credited to the enzymatic hydrolysis on phenolic acids coupled with the varietal specie of the cultivars. However, differences in thermal properties and the degree of crystallinity of sorghum, *Agrigreen* and *Babala* pearl millet on malted and fermented flours were related to the enzymatic changes. That is the endogenous microbes during malting and fermentation time that reduced the starch molecules to granules of amylose and amylopectin, which might affect the onset, melting point, end completion, gelatinisation time, enthalpy, the level of crystallinity and the functional groups due to the inability to break the granules of malted and fermented flours as a result of protein and starch-lipid complexes. The Fourier transform infrared (FTIR) spectroscopy of the malted and fermented *Agrigreen*, *Babala* and sorghum flours showed peaks in OH, carbonyl, amide and C–O bonding. X-ray diffractograms of the malted and fermented *Agrigreen*, *Babala* and sorghum flours exhibited high peak intensities, while the unprocessed flours showed diffused peaks. It could be concluded that processed on pearl millet cultivars and sorghum flour have an influence on the functional groups and the crystallinity level of the flours. The optimal processing time for malting and fermentation of colour were predicted as 50.69 and 39.38 h for *Agrigreen*, 54.40 and 63.30 for *Babala* and 49.90 and 54.61 for sorghum, while the optimum thermal properties for malting and fermentation time were 45.78 and 42.60 h for *Agrigreen*, 40.94 and 29.07 h for *Babala* and 34.83 and 36.33 h for sorghum, respectively with high desirability of 1.00. RSM was effective in optimising process parameters for *Agrigreen*, *Babala* of pearl millet cultivars and sorghum flours. Hence the optimal processing conditions obtained in this study could be used as a standard for the improvement of pearl millet varieties and sorghum flours for food processing companies.

## Declarations

### Author contribution statement

Gbeminiyi Olamit: Conceived and designed the experiments; Performed the experiments; Analyzed and interpreted the data; Contributed reagents, materials, analysis tools or data; Wrote the paper.

T. K. Takalani, D. Beswa, A. I. O. Jideani: Conceived and designed the experiments; Analyzed and interpreted the data; Contributed reagents, materials, analysis tools or data; Wrote the paper.

### Funding statement

This work was supported by the Research and Publications Committee (RPC), University of Venda, South Africa (SARDF/16/FST/04).

### Competing interest statement

The authors declare no conflict of interest.

### Additional information

No additional information is available for this paper.
